# Birth by caesarean section and school performance in Swedish adolescents- a population-based study

**DOI:** 10.1186/s12884-017-1304-x

**Published:** 2017-04-17

**Authors:** Eileen A. Curran, Louise C. Kenny, Christina Dalman, Patricia M. Kearney, John F. Cryan, Timothy G. Dinan, Ali S Khashan

**Affiliations:** 1 Department of Obstetrics and Gynaecology, The Irish Centre for Fetal and Neonatal Translational Research (INFANT), University College Cork, Cork, Ireland; 2 Department of Public Health Sciences, Division of Public Health Epidemiology, Karolinska Institutet, Stockholm, Sweden; 3 Department of Epidemiology and Public Health, University College Cork, Cork, Ireland; 4 Department of Anatomy and Neuroscience, APC Microbiome Institute, University College Cork, Cork, Ireland; 5 Department of Psychiatry, APC Microbiome Institute, University College Cork, Cork, Ireland

**Keywords:** Caesarean section: School performance, Cohort study, Quantile regression

## Abstract

**Background:**

Our objective was to assess the impact of obstetric mode of delivery, and in particular birth by Caesarean section (CS), on school performance in adolescents using a large, population-based cohort.

**Methods:**

We extracted data from the Swedish Medical Birth Register and National School Register. We included all live singleton births in Sweden from 1982–1995 (*n* = 1,489,925). School grades were reported on a scale from 0 to 320, scores less than 160 (i.e. “pass”) were considered to be “poor school performance.” Mode of delivery was categorised as: unassisted vaginal delivery (VD), assisted VD, elective CS and emergency CS. We measured the association between mode of delivery and “poor school performance” using logistic regression. We then used quantile regression to assess the association between mode of delivery and school performance across the distribution of scores. We adjusted for maternal age, parity, small and large for gestational age, gestational age, maternal country of birth, maternal depression, non-affective disorder or bipolar disorder, parental income at time of birth, and parental social welfare at time of birth. We also conducted sensitivity analyses to investigate the association further.

**Results:**

With logistic regression analysis, the adjusted odds ratio (aOR) of assisted VD and poor school performance, compared to unassisted VD, was 1.06 (95% CI: 1.03–1.08). For elective CS it was 1.06 (95% CI:1.03-1.09) and for emergency CS it was 1.12 (95% CI: 1.09–1.15). With quantile regression, assisted VD showed little difference in scores, when compared to unassisted VD, at any point across the distribution. Elective CS was associated with a 1–3 point decrease in scores, and emergency CS was associated with a 2–5 point decrease in scores.

**Conclusion:**

A slight association was found between birth by CS and school performance. However, the effect was quite small and given the complex nature of the relationship, should be interpreted with caution.

**Electronic supplementary material:**

The online version of this article (doi:10.1186/s12884-017-1304-x) contains supplementary material, which is available to authorized users.

## Background

Rates of Caesarean section (CS) are rising globally. A recent study including data from thirty one European countries, reported the median rate of CS in those countries to be 25%, [1] though this trend is by no means limited to Europe or other high-income countries. Another recent study reported that in 2010–2011, among the 21 countries included, the rate of CS in countries with a high Human Development Index (HDI) was 40.0%, in countries with moderate HDI it was 32.4% and in countries with low HDI it was 20.3% [2]. Given such a large and growing rate of CS, it is becoming of increasing interest to understand potential long-term effects of birth by CS, [3] as even a small increase in risk could potentially have a large impact globally.

It has been hypothesised that birth by CS leads to changes in psychological development, due to “early term” birth [4, 5] or alterations in microbiota or stress response [6]. In animal models, birth by CS and changes in microbiota have been associated with changes in behaviour, stress response, and anxiety [7]. Notably, these theories apply more to pre-labour or “elective” CS. Previous evidence suggests the association between CS and neurodevelopmental disorders in human populations may be primarily driven by confounding [8]. However, it is also possible that birth by CS may lead to sub-clinical changes in behavioural development, for example increased anxiety, that are not included in official diagnoses. One way to potentially assess the overall impact on psychological well-being is through school performance. School performance has been associated with several psychological outcomes including behavioural problems, [9] attention problems, [10] substance abuse, [10] and insomnia [11]. In addition, school performance as a teenager has been linked to psychological well-being as an adult, including depression and self-harm [12, 13]. Therefore, if CS has an impact on behavioural development, it may lead to changes in overall school performance.

Elective CS has been associated with delays in personal social skills and gross motor function at age 9 months, [14] and early term birth has been associated with increased special education requirements [4]. However, to our knowledge, no one has examined the potential association between obstetric mode of delivery, more specifically birth by CS, and school performance. To that end, the objective of the current study was to investigate the possible impact of birth by CS on school performance in a large, population-based Swedish cohort.

## Methods

### Data

#### Study Population

We included data from 4 Swedish Registers: Medical Birth Register, National School Register, Multi-Generation Register, and National Patient Register. Each resident of Sweden is given a personal identification number (PIN) which is the same in each of these registers, and can be used to link data across registers. The Medical Birth Register was established in 1973 and includes data on over 98% of all births in Sweden [15]. For our cohort, we included all live singleton births in the Swedish Medical Birth Register that occurred between 1982 and 1995. As both mode of delivery and school performance are likely to be highly correlated in multiple births, these were excluded. Variables detailing the timing of onset of labour and CS are available from 1982, thus marking the beginning of our cohort births. Ethical approval was obtained from the regional ethical research committee of Stockholm at Karolinska Institutet. Informed consent was waived by the ethics committee.

#### Exposure-obstetric mode of delivery

Obstetric mode of delivery, extracted from the Medical Birth Register, consisted of “unassisted vaginal delivery VD,” “assisted VD,” “elective CS” and “emergency CS”. Unassisted VD was defined as VD without the use of forceps or vacuum extraction, and assisted VD was VD with the use of forceps or vacuum extraction. Unassisted and assisted VD included both spontaneous and induced VD. Elective CS was defined as CS which started before onset of labour (as indicated on medical charts by water departure, bleeding or regular and sustained pain) and emergency CS was defined as CS which started after onset of labour.

#### Outcome-school performance

Data on school performance were extracted from the National School Register, which are available beginning in 1988. In Sweden, upon finishing the compulsory years of school (age 16), grades in 16 subjects are recorded. Starting in 1998, these grades were categorised into 4 levels for each subject: not pass (score of 0), pass (score of 10), pass with distinction (score of 15), and pass with great distinction (score of 20). This allowed for a maximum total score of 320 (i.e. a score of 20 in each of the 16 subjects). Prior to 1998 there was a different grading system, but as the oldest children in our cohort turned 16 in 1998 only the current method was included. Children that “drop out” of school before compulsory grading still technically graduate but are recorded as having received a total of 0 for their final grade, and are not able to continue on to high school. These children were included in our population and were recorded as having a total score of 0. Scores were assessed in both categorical and continuous (from 0 to 320 in jumps of 5) form [16, 17].

#### Co-variates

Based on previous literature and the use of a directed acyclic graph (DAG) [18] (Additional file 1: Figure S1), the following a priori co-variates were included in the analysis: maternal age at time of birth (<25 years, 25–34 years, 35–44 years, 45+ years), [16] birth order (first born), [16] small for gestational age (SGA), [16] large for gestational age (LGA) (defined as birth weight less or greater than 2 standard deviations from the mean for gestational age, respectively), gestational age (<37 weeks, 37, 38, 39, or 40 weeks, >40 weeks), [16] maternal country of birth (Swedish, other Nordic, other), [19] maternal depression, non-affective disorder, or bipolar disorder (never diagnosed, diagnosed before birth, diagnosed after birth), parental income at time of birth (in quintiles), and parental social welfare at time of birth (yes/no, note: available from 1983), [19] and parental highest education (pre-high school, high school, post-high school) [16, 19, 20].

Though not identified as confounders in the DAG, further co-variates that were identified based on previous literature were also assessed, including: year of birth, [20] year of school completion, smoking at time of first antenatal visit (none, 1–9 cigarettes/day, 10+ cigarettes/day), [16, 20] infant gender, [16, 19] Apgar score at 5 min (“low” [0–3], “intermediate” [4–6], “high” [7–10]), [16, 21] paternal country of birth (Swedish, other Nordic, other), [19] paternal depression, non-affective disorder, and bipolar disorder (never diagnosed, diagnosed before birth, diagnosed after birth), parental co-habitation at time of birth [19, 20].

All co-variates were tested individually in the logistic regression analysis to assess the potential impact on the association between mode of delivery and school performance. As no variable changed the estimate by more than 10% (only maternal age changed the estimate by more than 5%), only the variables decided on a priori were included in final analysis. Notably, parental education was considered an a priori variable, but was only available from 1990, and thus though it was tested individually, and as it had no impact on the estimate, was not included in the model. Distribution of each variable by mode of delivery is outlined in Table 1.

**Table 1:** Distribution of descriptive variables by mode of delivery

	Unassisted VD	Assisted VD	Elective CS	Emergency CS
Total Population	1036424 (84.51)	78441 (6.40)	52107 (4.25)	59357 (4.84)
Poor grade	138202 (13.33)	8880 (11.32)	7064 (13.56)	8203 (13.82)
Gender (Male)	522988 (50.46)	45361 (57.83)	26165 (50.21)	32357 (54.51)
Maternal age				
<20	30367 (2.93)	2679 (3.42)	698 (1.34)	1551 (2.61)
21–29	633991 (61.17)	50482 (64.36)	24004 (46.07)	33689 (56.76)
31–39	356370 (34.38)	24176 (30.82)	2486 (47.71)	22406 (37.75)
40+	15696 (1.51)	1104 (1.41)	2545 (4.88)	1711 (2.88)
First born child	599463 (57.84)	69919 (89.14)	31350 (60.16)	44922 (75.68)
Smoking at time of first antenatal visit				
None	704557 (67.98)	52772 (67.28)	35328 (67.80)	39483 (66.52)
1–9 Cigarettes/day	148178 (14.30)	11924 (15.20)	7746 (14.87)	9586 (16.15)
10+ Cigarettes/day	91075 (8.79)	6401 (8.16)	4979 (9.56)	5864 (9.88)
Missing	92614 (8.94)	7344 (9.36)	4054 (7.78)	4424 (7.45)
SGA	20383 (1.97)	2178 (2.78)	2969 (5.70)	5017 (8.45)
LGA	31455 (3.03)	2279 (2.91)	2996 (5.75)	2605 (4.39)
Parental education				
Pre-Highschool	34151 (3.30)	209 (2.67)	1878 (3.60)	2342 (3.95)
Highschool	232551 (22.44)	17107 (21.81)	11862 (22.76)	15568 (26.23)
Post-Highschool	151078 (14.58)	12916 (16.47)	8555 (16.42)	10506 (17.70)
Missing	9145 (0.88)	680 (0.87)	344 (0.66)	703 (1.18)
Born Prior to 1990	609499 (58.81)	45647 (58.19)	29468 (56.55)	30238 (50.94)
Parental income				
First	201292 (19.42)	8839 (11.27)	8943 (17.16)	9095 (15.32)
Second	210046 (20.27)	9836 (12.54)	10020 (19.23)	9407 (15.85)
Third	209679 (20.23)	13499 (17.21)	10215 (19.60)	10713 (18.05)
Fourth	204240 (19.71)	19267 (24.56)	10316 (19.80)	13159 (22.17)
Fifth	191366 (18.46)	25526 (32.54)	11730 (22.51)	15816 (26.65)
Missing	1981 (1.91)	1474 (1.88)	883 (1.69)	1167 (1.97)
Parental social welfare status				
Yes	84609 (8.16)	5597 (7.14)	4173 (8.10)	5490 (9.25)
No	864190 (83.38)	65913 (83.03)	44174 (84.78)	50000 (84.24)
Missing	19132 (1.85)	1416 (1.81)	858 (1.65)	1133 (1.91)
Born Prior to 1983	68493 (6.61)	515 (7.03)	2902 (5.57)	2734 (4.61)
Apgar Score (5 min)				
Low (0–3)	1484 (0.14)	241 (0.31)	141 (0.27)	417 (0.70)
Intermediate (4–6)	3375 (0.33)	1501 (1.91)	479 (0.92)	1836 (3.09)
High (7–10)	1004980 (96.97)	75464 (96.20)	50105 (96.16)	55775 (93.97)
Missing	26585 (2.57)	1235 (1.57)	1382 (2.65)	1329 (2.24)
Gestational age				
<37 Weeks	39384 (3.80)	1971 (2.51)	5941 (11.40)	10820 (18.23)
37 Weeks	48430 (4.67)	2546 (3.25)	5923 (11.37)	4508 (7.59)
38 Weeks	122702 (11.84)	6707 (8.55)	25006 (47.99)	7487 (12.61)
39 Weeks	255027 (24.61)	15235 (19.42)	9990 (19.17)	8416 (14.18)
40 Weeks	311130 (30.02)	23094 (29.44)	2714 (5.21)	11056 (18.63)
>40 Weeks	257116 (24.81)	28678 (36.56)	2400 (4.61)	16892 (28.46)
Missing	2635 (0.25)	210 (0.27)	133 (0.26)	178 (0.30)
Maternal Depression				
Never Diagnosed	952661 (91.92)	71714 (91.42)	46936 (90.08)	53615 (90.33)
Diagnosed before birth	6051 (0.58)	457 (0.58)	584 (1.12)	540 (0.91)
Diagnosed after birth	77712 (7.50)	6270 (7.99)	4587 (8.80)	5202 (8.76)
Maternal Non-affective Disorder				
Never Diagnosed	1026908 (99.08)	77638 (99.98)	51460 (98.76)	58644 (98.82)
Diagnosed before birth	2392 (0.23)	227 (0.29)	209 (0.40)	201 (0.34)
Diagnosed after birth	7124 (0.69)	576 (0.73)	438 (0.84)	501 (0.84)
Maternal Bipolar Disorder				
Never Diagnosed	1026027 (99.00)	77649 (98.99)	51507 (98.85)	58702 (98.90)
Diagnosed before birth	818 (0.08)	82 (0.10)	80 (0.15)	61 (0.10)
Diagnosed after birth	9579 (0.92)	710 (0.91)	520 (1.00)	594 (1.00)
Paternal Depression				
Never Diagnosed	983156 (94.86)	74367 (94.81)	49255 (94.53)	56114 (94.54)
Diagnosed before birth	4697 (0.45)	309 (0.39)	309 (0.59)	325 (0.55)
Diagnosed after birth	48571 (4.69)	3765 (4.80)	2543 (4.88)	2918 (4.92)
Paternal Non-affective Disorder				
Never Diagnosed	1028187 (99.21)	77816 (99.20)	51705 (99.23)	58823 (99.10)
Diagnosed before birth	2360 (0.23)	171 (0.22)	134 (0.26)	167 (0.28)
Diagnosed after birth	5877 (0.57)	454 (0.58)	268 (0.51)	367 (0.62)
Paternal Bipolar Disorder				
Never Diagnosed	1029320 (99.31)	77915 (99.33)	51703 (99.22)	58919 (99.26)
Diagnosed before birth	805 (0.08)	65 (0.08)	68 (0.13)	43 (0.07)
Diagnosed after birth	6299 (0.61)	461 (0.59)	336 (0.63)	395 (0.67)
Co-habitating with child's father				
Yes	894777 (86.33)	66362 (84.60)	45489 (87.30)	50855 (85.68)
No	47235 (4.56)	4655 (5.93)	2389 (4.58)	3464 (5.84)
Missing	94412 (9.11)	7424 (9.46)	4229 (8.12)	5038 (8.49)
Maternal Country of Birth				
Sweden	917862 (88.56)	69758 (88.93)	45638 (87.59)	51349 (86.51)
Other Nordic	41385 (3.99)	2935 (3.74)	2327 (4.47)	2526 (4.26)
Other	77170 (7.45)	5747 (7.33)	4141 (7.95)	5482 (9.24)
Missing	7 (0.00)	1 (0.00)	1 (0.00)	0 (0.00)
Paternal Country of Birth				
Sweden	905824 (87.40)	68936 (87.88)	45771 (87.84)	51084 (86.06)
Other Nordic	34953 (3.37)	2445 (3.12)	1910 (3.67)	2153 (3.63)
Other	90567 (8.74)	6485 (8.27)	4155 (7.97)	5713 (9.62)
Missing	5080 (0.49)	575 (0.70)	271 (0.52)	407 (0.69)
Natural science poor performance	298903 (28.43)	21026 (26.51)	15266 (28.86)	17513 (29.09)
Civics poor performance	408082 (38.81)	49818 (37.18)	32022 (39.47)	36182 (39.91)
Sports poor performance	70358 (6.79)	4365 (5.56)	3611 (6.93)	4048(6.82)
Arts poor performance	62182 (5.91)	3937 (4.96)	3223 (6.09)	3644 (6.05)
Swedish poor performance	68154 (6.48)	4196 (5.29)	3467 (6.55)	4297 (7.14)

*Abbreviations*: *VD* vaginal delivery, *CS* Caesarean section, *SGA* small for gestational age, *LGA* large for gestational age

### Statistical analysis

#### Logistic regression

For the logistic regression, we considered “poor school performance” to be a total score of less than160, [16, 17, 22] meaning the individual did not have an average of at least 10 (i.e. “pass”) for the 16 subjects. In Sweden, scores are assigned by teachers rather than a standardised test, and thus standards for a particular grade could vary school-to-school. To account for this, we used mixed effects modelling with a random intercept for school ID.

#### Quantile regression

The data on school performance have been previously reported to be highly skewed [16, 20]. We used quantile regression to analyse school performance in its continuous form. Quantile regression is similar to an ordinary least squares (OLS) model, except the model regresses on the quantile of interest (such as the median), instead of the mean. Quantile regression also does not require an assumption of normality or equal variance, and allows for assessment across the distribution (i.e. at every quantile). In this way we were able to determine if there was an effect of mode of delivery across the distribution of scores (for example, a possible effect only on the high or low scores), rather than an effect only on passing scores as seen with logistic regression. We plotted quantile regression coefficients for every fifth quantile from the 5th to the 95th using the kernel-based method for estimating standard errors [23]. We also looked at coefficient estimates for specifically the 5th, 25th, 50th, 75th, and 95th quantiles. In adjusted analysis we included the same co-variates as the fully adjusted logistic regression model.

#### Additional analyses

We conducted several sub-group analyses. In the logistic regression, we restricted to births from 1990 onwards (the year data on parental education became available), and assessed the association with and without adjustment for parental education. We assessed the association only among male babies. We also excluded children born through a secondary CS (children born by CS whose mothers’ had previously given birth through CS), and children with a low Apgar score at 5 min. Though the vast majority of the population finishes compulsory years of school at age 16 (95%), there are some students who finish younger or older. To that end, we also restricted the population to those who were 16 at the time they finished compulsory school to determine what effect age may have had on school performance. To account for potential clustering of academic performance within families, we restricted the population to one-child families and first born children. We then repeated overall analysis with a random intercept for maternal ID instead of school ID. For both logistic and quantile regression we conducted sensitivity analyses by excluding children who received a “0” as a grade (i.e. children who did not complete the compulsory years of schooling).

Additionally, we conducted logistic regression to assess the association between birth by CS and school performance in five subject categories: [12] natural sciences (biology, chemistry and physics), social sciences (geography, religion, history and society knowledge), arts (art and handicraft), sports, and Swedish. An average below “pass” (10 points per subject) was considered poor performance in each category. Similar to overall school performance analysis, sub-group analyses were conducted where children who were recorded as a “0” in any subject were excluded from that category.

The logistic regression analysis was conducted in SAS v9.3 (Cary, N.C) using PROC GLIMMIX [24] and quantile regression analysis was conducted in R v3.2.2 using the QUANTREG package [23]. Missing data were addressed using the missing indicator method, with a category for each variable used to indicate “missing” status [25].

## Results

### Descriptive statistics

**Fig. 1:**
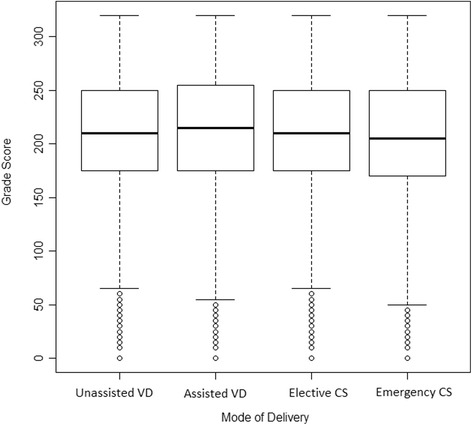
Distribution of school grades by mode of delivery. *Abbreviations*: OR: odds ratio, VD: vaginal delivery, CS: Caesarean section. *Interpretation: the *bold lines* represent the median (50th percentile), *lines* at the top and bottom of each box represent the 25th and 75th percentiles, respectively. Dotted lines extend to the most extreme data point that is within 1.5 times the inter-quartile range of the box. Circles represent outliers, or data points outside this range

There were 1,489,925 live births in Sweden from 1982–1995. We excluded 34,199 (2.3%) multiple births, and 52,562 (3.5%) births missing mode of delivery, leaving a total of 1,403,164 births. Of these, 176,836 (12.6%) did not have grades recorded in the National School Register. Possible reasons for not being recorded in the National School Register include death, emigration, and attendance at a specialised school (for example if the child had a disability) [21]. There were 143,740 missing that were born through unassisted VD (12.2%), 11,538 from assisted VD (12.8%), 9,615 from elective CS (15.6%) and 11,943 from emergency CS (16.8%). Our final cohort consisted of 1,243,876 children. Of these, 1,036,424 were born by unassisted VD (84.5%), 78,441 by assisted VD (6.4%), 42,107 by elective CS (4.3%) and 59,357 by emergency CS (4.8%) (Table 1). 162,349 (13.2%) children had poor school performance. Ages at time of grading ranged from 14–21, with 95% of children receiving grades at 16 years of age. The median score was 210 (Inter-quartile range: 175–250) (Fig. 1).

### Logistic regression

In unadjusted analysis, the OR for assisted VD compared to unassisted VD was 0.84 [95% CI:0.82–0.86] (Table 2). After adjustment it was 1.06 [95% CI:1.03–1.08]. For elective CS the unadjusted OR was 1.05 [95% CI: 1.03–1.08] and the adjusted OR was 1.06 [95% CI:1.03–1.09]. For emergency CS the unadjusted OR was 1.05 [95% CI:1.02–1.07] and increased to 1.12 [95% CI:1.09–1.15] after adjustment. Adjusting for birth year, year of receiving grades, and parental education (for those born after 1990) had no effect on results (Additional file 2: Table S1 and Additional file 3: Table S2). Results were consistent among male babies, children born in Stockholm County, children who were 16 years old when they received grades, children without a low Apgar score, and children with a score above 0 (Additional file 4: Table S3). Similarly, there seemed to be no family effect seen by creating a random intercept for maternal ID, or restricting the population based on family size and birth order (Data not shown, available on request).

**Table 2:** Association between mode of delivery and poor school performance

	Exposed Cases	Unadjusted OR (95% CI)	Adjusted OR (95% CI)
Unassisted VD	138202	Ref	Ref
Assisted VD	8880	0.84 (0.82-0.86)	1.06 (1.03-1.08)
Elective CS	7064	1.05 (1.03-1.08)	1.06 (1.03-1.09)
Emergency CS	8203	1.05 (1.02-1.07)	1.12 (1.09-1.15)

*Abbreviations*: *OR* odds ratio, *VD* vaginal delivery, *CS* Caesarean section

When school performance was divided into subjects, the association was similar to the overall association between birth by CS and poor school performance (Additional file 5: Table S4).

### Quantile regression

**Fig. 2:**
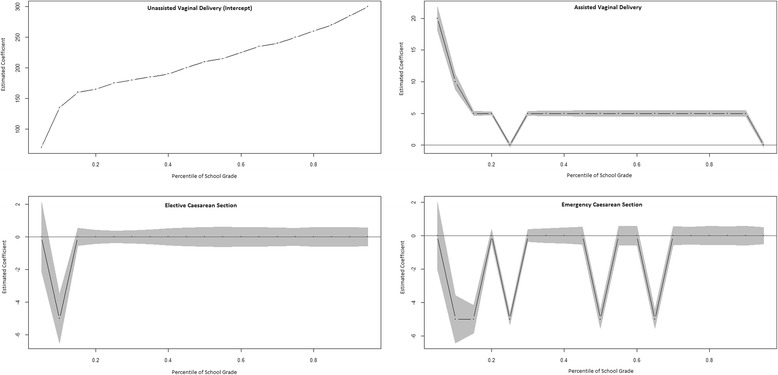
Unadjusted quantile regression modelling the association between mode of delivery and school performance. *Interpretation: the coefficient values for unassisted vaginal delivery correspond to the school grade at that percentile. Estimated coefficients for other modes of delivery correspond to the estimated difference in score at each percentile. Grey area indicates 95% confidence intervals. *Note*: Percentiles are recorded as proportions. For example, 0.2 corresponds to the 20th percentile etc

**Fig. 3:**
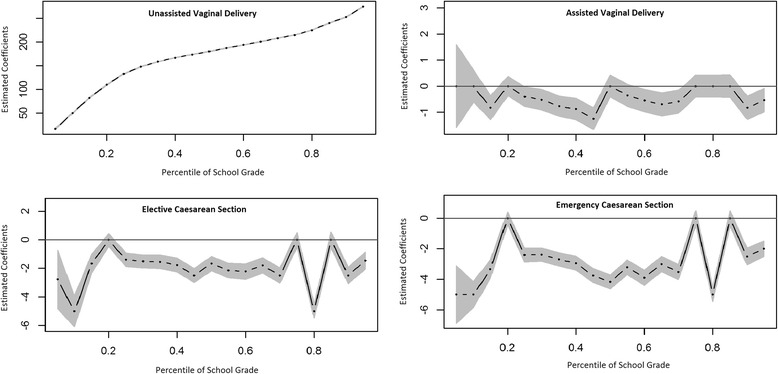
Adjusted quantile regression modelling the association between mode of delivery and school performance. *Interpretation: the coefficient values for unassisted vaginal delivery correspond to the predicted school grade at that percentile, given reference value for all co-variates. Estimated coefficients for other modes of delivery correspond to the estimated difference in score at each percentile. Grey area indicates 95% confidence intervals

There was little difference in the distribution of grades by mode of delivery (Fig. 1). The plots of estimates across the distribution, as well an explanation for how to interpret them, is shown in Fig. 2. Scores for children born through assisted VD were slightly higher than unassisted VD, especially at lower quantiles. There was no association with elective or emergency CS. After adjustment, there was no association between assisted VD and school performance (Fig. 3 and Additional file 6: Figure S2). Elective and emergency CS were associated with a slight decrease (2–5 points) in scores across the distribution.

In unadjusted analysis, the 5th, 25th, 50th, 75th and 95th percentiles for unassisted VD were 70, 175, 210, 250 and 300, respectively. Assisted VD was 20 points higher for the 5th percentile, and 5 points higher for the 50th and 75th percentiles. Elective CS was the same as unassisted VD at all five percentiles. Emergency CS was 5 points lower than unassisted VD at the 25th and 50th percentiles. In adjusted analysis, the 5th, 25th, 50th, 75th, and 95th percentiles for unassisted VD were 17.2, 132.8, 180, 215, and 274.7, respectively. Assisted VD was largely equal to unassisted VD. Elective CS showed a slight decrease (ranging from −1.4 to −2.78) in scores across the distribution, with the exception of the 75th percentile where it was equal. Similarly, emergency CS also showed a slight decrease in scores (ranging from −2 to −5) with the exception of the 75th percentile, which showed no change. Excluding scores of 0 did not change results (Data not shown, available on request).

## Discussion

### Main findings

We assessed the impact of obstetric mode of delivery, and in particular birth by CS, on school performance at age 16 using a large, population-based cohort. Two separate analyses were conducted, logistic and quantile regression, assessing school performance in both dichotomous and continuous form. There was a slight association between birth by CS and a reduction in school performance in both analyses. In logistic regression, elective CS was significantly associated with increased likelihood of poor school performance. However, children born by CS were only 6% more likely to receive a poor grade when compared to children born through unassisted VD. The association between emergency CS and poor school performance was somewhat stronger but still small (OR = 1.12, [95% CI:1.09–1.15]). With quantile regression analysis, there again appeared to be a slight association between birth by CS and school performance, primarily in adjusted results. Children born by elective or emergency CS had a 1–5 point decrease in score across the distribution, translating to a 0.31–1.56% decrease.

### Interpretation

There are several possible explanations for an observed association between birth by CS and a small reduction in school performance. A range of characteristics influence school performance including not only behaviour [9] and personality, [26] but also cognitive ability, [26] and external factors such as ethnic diversity in the district [19]. It is possible that rather than having an effect across this wide range of factors and behaviours, birth by CS is having an effect on only one aspect, such as anxiety. Another potential explanation is that this result is being driven by confounding, such as confounding by indication or residual confounding. Confounding by indication occurs when an outcome is causally associated with an indication for the exposure of interest [27]. For example, foetal distress and maternal anxiety may be indications for emergency or elective CS, [28] and may also have an impact on school performance [29, 30], leading to a non-causal association between CS and poor school performance. Additionally, the association could be driven by residual confounding [27]. The relationship between pre and perinatal risk factors and psychological development is complex, and it can be difficult to rule out the effect of difficult to measure confounders, such as social adversity [31]. Regardless of what is driving the association, the decrease in score is very slight. A previous study on this population reported comparable effect sizes for the association between current asthma, rhinitis, eczema and school performance (change in mean score ranging from −3.1 to 4.1) and similarly concluded that though there were statistically significant associations they were likely not clinically meaningful or causally associated [32]. For comparison, the same study reported that severe nasal symptoms is associated with a 12.1 point decrease in mean grade, [32] and another study reported consumption of fish at least once a week is associated with a 14.5–19.9 point increase in mean grade [33].

### Strengths and limitations

The strengths of the present study include the use of population-based registries, limiting selection, information bias and measurement error, as we were able to include the entire population and received data from official records. Also, due to the extensive nature of these registries, we were able to assess the effect of a wide variety of co-variates and potential confounders including not only obstetric information, but also demographic and socio-economic factors. Additionally, due to the grading system in Sweden and the use of quantile regression, we were able to assess the impact of mode of delivery across a range of school grades, rather than merely assessing the likelihood of a “passing” grade.

The present study also has several limitations. First, we had no data on breast feeding, which has been linked to both mode of delivery [34] and school performance [35]. However, Sweden has a very high rate of breast feeding and close to 100% of Swedish-born children have ever been breast fed [36]. Additionally, as breast feeding may be affected by mode of delivery it is more likely to be a mediator rather than a confounder in this situation. Second, a range of factors affect school performance, and we cannot rule out a potential effect on more specific outcomes, such as anxiety, disruptive behaviour, or cognition. Previous results would indicate that mode of delivery does not have an impact on childhood neurodevelopment, [8] but results on behavioural difficulties are conflicting [14, 37]. Finally, it is worth noting that birth by CS in Sweden may not be representative. Access to medical care in Sweden is egalitarian, and the associations between social class and CS seen in other countries are not as prevalent [38]. Additionally, Sweden has a very low rate of birth by CS compared to other European countries, [1] and it is probable that we had a low incidence of non-medically indicated CS. Though we did not have access to information on indications for CS, we were able to separate pre- and post-labour CS, which had no impact on results.

## Conclusions

The present study used two analysis methods, adjusted for a wide variety of potential confounders, and conducted several sensitivity analyses to further investigate a potential association. With these robust analysis methods, we have concluded there is a slight association between birth by CS and poor school performance. Given the complex nature of the relationship between perinatal risk factors, such as birth by CS, and development, this small association should be interpreted with caution.

## Additional files

Additional file 1: Figure S1. Proposed directed acyclic graph (DAG) describing the association between birth by Caesarean section and poor school performance. Dark grey corresponds to variables which were measured, light grey corresponds to variables which were not measured or are difficult to fully quantify. (JPG 340 kb)

Additional file 2: Table S1. Effect of birth year and year of grading on the association between mode of delivery and poor school performance. (DOCX 15 kb)

Additional file 3: Table S2. Effect of parental education on the association between mode of delivery and poor school performance among children born in 1990 or later. (DOCX 14 kb)

Additional file 4: Table S3. Sensitivity analyses examining the effect of gender, county of birth, age at grading and Apgar score on the association between mode of delivery and poor school performance. (DOCX 15 kb)

Additional file 5: Table S4. The association between mode of delivery and poor school performance by subject. (DOCX 17 kb)

Additional file 6: Figure S2. Complete model of the adjusted association between mode of delivery and school performance. (TIF 1994 kb)

## Abbreviations

aOR: Adjusted odds ratio
CS: Cesarean section
DAG: Directed acyclic graph
HDI: Human development index
LGA: Large for gestational age
PIN: Personal identification number
SGA: Small for gestational age
VD: Vaginal delivery
