# Supplementary figures and images for: Birth by caesarean section and school performance in Swedish adolescents- a population-based study
Source: BMC Pregnancy Childbirth. 2017 Apr 17;17:121. doi: 10.1186/s12884-017-1304-x (PMC5392943; doi:10.1186/s12884-017-1304-x)

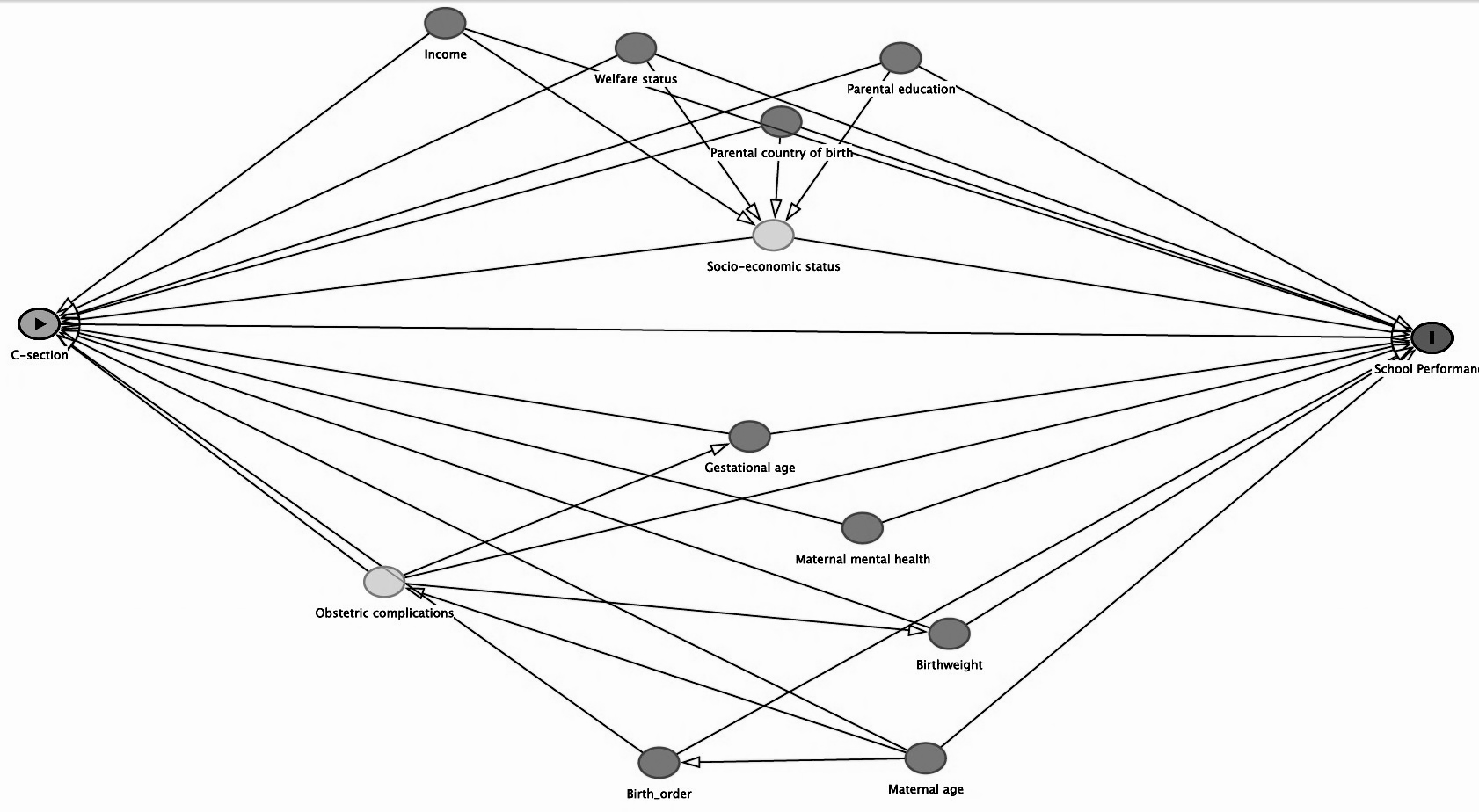

Supplement: Supplementary file 1 — Proposed directed acyclic graph (DAG) describing the association between birth by Caesarean section and poor school performance. Dark grey corresponds to variables which were measured, light grey corresponds to variables which were not measured or are difficult to fully quantify. (JPG 340 kb) [file 12884_2017_1304_MOESM1_ESM.jpg]

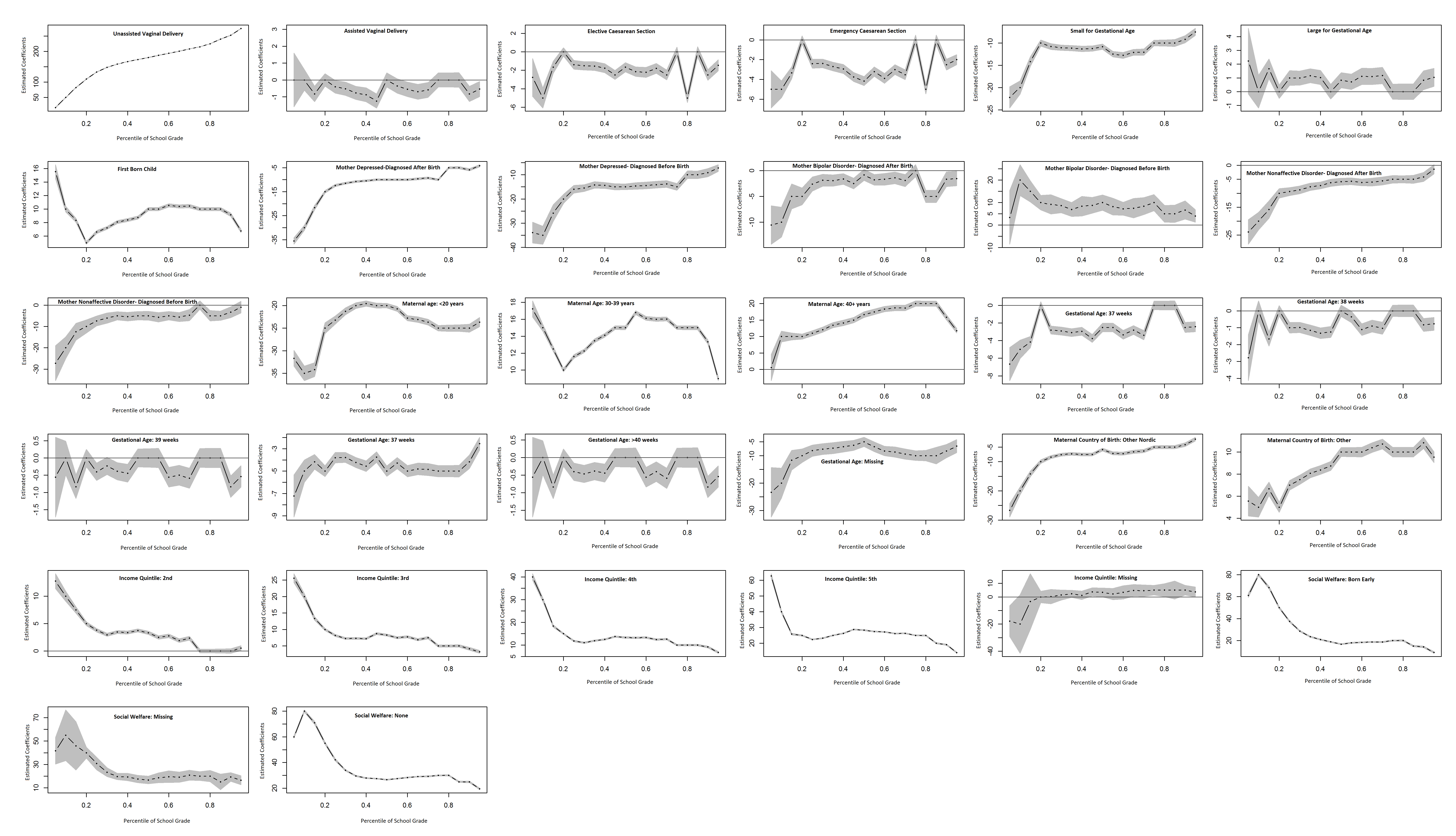

Supplement: Supplementary file 6 — Complete model of the adjusted association between mode of delivery and school performance. (TIF 1994 kb) [file 12884_2017_1304_MOESM6_ESM.tif]
