# Supplementary material for: Birth by caesarean section and school performance in Swedish adolescents- a population-based study
Source: BMC Pregnancy Childbirth. 2017 Apr 17;17:121. doi: 10.1186/s12884-017-1304-x (PMC5392943; doi:10.1186/s12884-017-1304-x)
Supplement: Supplementary file 2 — Effect of birth year and year of grading on the association between mode of delivery and poor school performance. (DOCX 15 kb) [file 12884_2017_1304_MOESM2_ESM.docx]

Additional file 2: Table S1. Effect of birth year and year of grading on the association between mode of delivery and poor school performance

|  | **Unadjusted**  **OR (95% CI)** | | | **Adjusted for birth year**  **OR (95% CI)** | | | **Adjusted for year of grading**  **OR (95% CI)** | | |
| --- | --- | --- | --- | --- | --- | --- | --- | --- | --- |
| Unassisted VD | Ref |  |  | Ref |  |  | Ref |  |  |
| Assisted VD | 0.84 | (0.82- | 0.86) | 0.84 | (0.82- | 0.86) | 0.84 | (0.82- | 0.86) |
| Elective CS | 1.05 | (1.03- | 1.08) | 1.05 | (1.03- | 1.08) | 1.05 | (1.03- | 1.08) |
| Emergency CS | 1.05 | (1.02- | 1.07) | 1.05 | (1.03- | 1.08) | 1.05 | (1.03- | 1.08) |

*Abbreviations*: OR-Odds ratio; VD-vaginal delivery; CS-Caesarean section
