# Supplementary material for: Birth by caesarean section and school performance in Swedish adolescents- a population-based study
Source: BMC Pregnancy Childbirth. 2017 Apr 17;17:121. doi: 10.1186/s12884-017-1304-x (PMC5392943; doi:10.1186/s12884-017-1304-x)
Supplement: Supplementary file 3 — Effect of parental education on the association between mode of delivery and poor school performance among children born in 1990 or later. (DOCX 14 kb) [file 12884_2017_1304_MOESM3_ESM.docx]

Additional file 3: Table S2. Effect of parental education on the association between mode of delivery and poor school performance among children born in 1990 or later

|  | **Unadjusted 1990**  **OR (95% CI)** | | | **Adjusted for parental education 1990**  **OR (95% CI)** | | | **Adjusted for co-variates except parental education 1990**  **OR (95% CI)** | | | **Adjusted including parental education 1990**  **OR (95% CI)** | | |
| --- | --- | --- | --- | --- | --- | --- | --- | --- | --- | --- | --- | --- |
| Unassisted VD | Ref |  |  | Ref |  |  | Ref |  |  | Ref |  |  |
| Assisted VD | 0.82 | (0.79- | 0.85) | 0.85 | (0.81- | 0.88) | 1.05 | (1.01- | 1.09) | 1.05 | (1.01- | 1.09) |
| Elective CS | 1.11 | (1.06- | 1.15) | 1.12 | (1.08- | 1.17) | 1.09 | (1.04- | 0.13) | 1.07 | (1.02- | 1.17) |
| Emergency CS | 1.01 | (0.97- | 1.04) | 1.00 | (0.97- | 1.04) | 1.12 | (1.08- | 1.16) | 1.09 | (1.05- | 1.13) |

*Abbreviations*: OR-Odds ratio; VD-vaginal delivery; CS-Caesarean section
