# Supplementary material for: Birth by caesarean section and school performance in Swedish adolescents- a population-based study
Source: BMC Pregnancy Childbirth. 2017 Apr 17;17:121. doi: 10.1186/s12884-017-1304-x (PMC5392943; doi:10.1186/s12884-017-1304-x)
Supplement: Supplementary file 4 — Sensitivity analyses examining the effect of gender, county of birth, age at grading and Apgar score on the association between mode of delivery and poor school performance. (DOCX 15 kb) [file 12884_2017_1304_MOESM4_ESM.docx]

Additional file 4: Table S3. Sensitivity analyses examining the effect of gender, county of birth, age at grading and Apgar score on the association between mode of delivery and poor school performance

|  | **Total Population**  **OR (95% CI)** | | | **Among Male Babies**  **OR (95% CI)** | | | **Stockholm County**  **OR (95% CI)** | | | **16 Year Olds**  **OR (95% CI)** | | | **No low Apgar Score**  **OR (95% CI)** | | |
| --- | --- | --- | --- | --- | --- | --- | --- | --- | --- | --- | --- | --- | --- | --- | --- |
| Unassisted VD | Ref |  |  | Ref |  |  | Ref |  |  | Ref |  |  | Ref |  |  |
| Assisted VD | 1.06 | (1.03- | 1.08) | 1.03 | (1.00- | 1.06) | 1.07 | (1.02- | 1.13) | 1.06 | (1.03- | 1.09) | 1.06 | (1.03- | 1.08) |
| Elective CS | 1.06 | (1.03- | 1.09) | 1.06 | (1.02- | 1.09) | 1.03 | (0.96- | 1.10) | 1.05 | (1.02- | 1.08) | 1.06 | (1.03- | 1.09) |
| Emergency CS | 1.12 | (1.09- | 1.15) | 1.11 | (1.07- | 1.14) | 1.08 | (1.02- | 1.14) | 1.10 | (1.07- | 1.13) | 1.11 | (1.08- | 1.14) |

*Abbreviations*: OR-Odds ratio; VD-vaginal delivery; CS-Caesarean section
