# Supplementary material for: Birth by caesarean section and school performance in Swedish adolescents- a population-based study
Source: BMC Pregnancy Childbirth. 2017 Apr 17;17:121. doi: 10.1186/s12884-017-1304-x (PMC5392943; doi:10.1186/s12884-017-1304-x)
Supplement: Supplementary file 5 — The association between mode of delivery and poor school performance by subject. (DOCX 17 kb) [file 12884_2017_1304_MOESM5_ESM.docx]

Additional file 5: Table S4. The association between mode of delivery and poor school performance by subject

|  | Unadjusted Assisted VD  OR (95%CI) | | | Unadjusted Elective CS  OR (95%CI) | | | Unadjusted Emergency CS  OR (95%CI) | | | Adjusted Assisted VD  OR (95%CI) | | | Adjusted Elective CS  OR (95%CI) | | | Adjusted Emergency CS  OR (95%CI) | | |
| --- | --- | --- | --- | --- | --- | --- | --- | --- | --- | --- | --- | --- | --- | --- | --- | --- | --- | --- |
| Natural science poor performance | 0.87 | (0.85- | 0.89) | 1.02 | (0.99- | 1.04) | 0.99 | (0.97- | 1.01) | 1.02 | (1.00- | 1.04) | 1.03 | (1.00- | 1.05) | 1.03 | (1.01- | 1.06) |
| Excluding ”0” | 0.82 | (0.80- | 0.84) | 1.04 | (1.01- | 1.07) | 1.01 | (0.98- | 1.04) | 1.04 | (1.01- | 1.07) | 1.06 | (1.03- | 1.09) | 1.08 | (1.05- | 1.11) |
| Civics poor performance | 0.91 | (0.89- | 0.93) | 1.02 | (0.99- | 1.04) | 1.03 | (1.01- | 1.06) | 1.02 | (1.00- | 1.04) | 1.01 | (0.99- | 1.04) | 1.05 | (1.03- | 1.08) |
| Excluding ”0” | 0.83 | (0.80- | 0.86) | 1.02 | (0.99- | 1.06) | 1.02 | (0.98- | 1.05) | 1.04 | (1.01- | 1.08) | 1.03 | (1.00- | 1.07) | 1.09 | (1.05- | 1.12) |
| Sports poor performance | 0.82 | (0.80- | 0.85) | 1.05 | (1.01- | 1.09) | 1.01 | (0.98- | 1.04) | 1.00 | (0.96- | 1.03) | 1.05 | (1.01- | 1.09) | 1.06 | (1.01- | 1.09) |
| Excluding ”0” | 0.82 | (0.80- | 0.85) | 1.05 | (1.01- | 1.09) | 1.01 | (0.98- | 1.04) | 1.00 | (0.96- | 1.03) | 1.05 | (1.01- | 1.09) | 1.06 | (1.03- | 1.10) |
| Arts poor performance | 0.84 | (0.81- | 0.87) | 1.05 | (1.01- | 1.10) | 1.02 | (0.99- | 1.06) | 1.03 | (0.99- | 1.07) | 1.06 | (1.01- | 1.10) | 1.08 | (1.04- | 1.12) |
| Excluding ”0” | 0.84 | (0.81- | 0.87) | 1.05 | (1.01- | 1.10) | 1.03 | (0.99- | 1.06) | 1.03 | (0.99- | 1.07) | 1.05 | (1.01- | 1.10) | 1.08 | (1.04- | 1.12) |
| Swedish poor performance | 0.82 | (0.80- | 0.85) | 1.04 | (1.00- | 1.08) | 1.11 | (1.07- | 1.15) | 1.04 | (1.00- | 1.08) | 1.05 | (1.01- | 1.10) | 1.17 | (1.13- | 1.21) |
| Excluding ”0” | 0.86 | (0.83- | 0.90) | 1.10 | (1.05- | 1.15) | 1.14 | (1.09- | 1.19) | 1.08 | (1.03- | 1.13) | 1.11 | (1.06- | 1.17) | 1.19 | (1.14- | 1.25) |

*Abbreviations*: VD-vaginal delivery; CS-Caesarean section
